# Characteristics and outcomes of unplanned intensive care unit admission after general anesthesia

**DOI:** 10.1186/s12871-022-01729-y

**Published:** 2022-06-20

**Authors:** Nobuyuki Katori, Kentaro Yamakawa, Kosuke Yagi, Yoshihiro Kimura, Mayuko Doi, Shoichi Uezono

**Affiliations:** Department of Anesthesiology, The Jikei University School of Medicine, 3-25-8 Nishishinbashi, Minatoku, Tokyo 105-8461 Japan

**Keywords:** Intensive care unit, Post-anesthesia care unit, Unplanned admission, Prolonged stay, Non-cardiac surgery

## Abstract

**Background:**

Unplanned ICU admission after surgery has been validated as a measure of a quality indicator of perioperative management because it may put surgical patients at risk of increased morbidity and mortality. Postoperative unscheduled admission to the ICU is usually determined either in the post-anesthesia care unit (PACU) or in the general surgical ward; however, it could be expected patient outcomes after ICU admission would be affected by the circumstances. The purpose of this retrospective observational study was to investigate the clinical characteristics and the outcome of unplanned admission to the ICU directly from the PACU or from the ward within 7 days after PACU discharge.

**Methods:**

Forty-three thousand, five hundred fifty-three patients admitted to the PACU after general anesthesia were included in the study. Unplanned ICU admission was defined as the admission which was not anticipated preoperatively but was due to adverse events in the PACU (PACU group) or the ward after discharge from the PACU (Ward group). The following parameters were compared between the groups: patient characteristics, surgical characteristics, length of ICU and hospital stay, the principal adverse event for ICU admission, treatments in the ICU, and in-hospital mortality. The primary outcome was in-hospital mortality and the second was the length of ICU and hospital stay.

**Results:**

Among 43,553 patients, 109 patients underwent unplanned ICU admission directly from the PACU (*n*= 73, 0.17%) or subsequently from the ward (*n*= 36, 0.08%). The length of both ICU and hospital stay was significantly longer in the Ward group than in the PACU group (1.4 and 19 days vs. 2.5 and 39 days, respectively). There was no significant difference in in-hospital mortality between the groups (4.1% vs. 8.3%, respectively).

**Conclusions:**

The incidence of unplanned ICU admission after PACU stay was low, however, delayed admission to the ICU from the ward may prolong the length of both ICU and hospital stay compared to those directly from the PACU.

## Backgrounds

The admission to the intensive care unit (ICU) of the surgical patient is evaluated preoperatively considering the severity of the patient’s comorbidities and the risk of the surgical procedure. However, some patients may need unplanned ICU admission because of unexpected postoperative adverse events or abrupt deterioration. Unplanned ICU admission after surgery has been validated as a measure of patient safety and a quality indicator of perioperative management because it may put surgical patients at risk of increased morbidity and mortality [1, 2].

Indeed, postoperative unscheduled admission to the ICU is usually determined either in the post-anesthesia care unit (PACU) or in the general surgical ward, it could be expected patient outcomes after ICU admission would be affected by the circumstances: the timing when adverse events are recognized, the place where adverse events happen, and who decides ICU admission. Thus, we considered the investigation of the clinical features of adverse events leading to unplanned ICU admission would contribute to the improvement of postoperative care either in the PACU or the ward. The aim of this study was to investigate patient characteristics and outcomes of unplanned ICU admission from the PACU or the ward within 7 days after PACU discharge.

## Methods

This retrospective observational study was reviewed and approved by the Ethics committee of the Jikei University, Tokyo, Japan. The need for informed and written consent was waived because of the nature of the retrospective study by Clinical Research Review Board of the Jikei University. This single-institution retrospective study was performed in accordance with the Declaration of Helsinki and Clinical Trials Act established by the Japanese Ministry of Health, Labour and Welfare.

Patients who underwent general anesthesia and were also monitored postoperatively in the PACU between July 2009 and December 2018 at a 1075-bed university hospital with a 20-bed ICU were included in the study. Among these patients, those who were admitted to the ICU unexpectedly within seven days after the surgery were retrospectively assessed using the electrical records. Unplanned ICU admission was defined as the admission which was not anticipated preoperatively but was due to adverse events in the PACU (PACU group) or the ward after discharge from the PACU (Ward group). We excluded the following patients from the analysis: patients younger than 20 years of age, patients who were already in the ICU before surgery, and patients who were transferred to the ICU directly from the operating room (OR).

At our institution, there are no specific criteria for ICU admissions after surgery. The decision of postoperative ICU admission is made preoperatively on a case-by-case basis by the surgical team in consultation with the anesthesia team in the OR. Patients who are scheduled for postoperative ICU care and those who are deemed worth the ICU care owing to unexpected deterioration or adverse events during the surgery are transferred directly to the ICU from the OR, while the other patients are admitted to the PACU from the OR and then transferred to the ward after the evaluation according to the PACU discharge criteria base on the modified Aldrete score [3].

The following data were collected for the analysis: patient characteristics including gender, age, body mass index, and American Society of Anesthesiologists physical status (ASA-PS); surgical procedures performed; length of surgery and anesthesia; length of PACU stay; the principal adverse event for ICU admission; treatments in the ICU; length of stay in the ICU and hospital; and in-hospital mortality. The primary outcome was in-hospital mortality and the second was the length of ICU and hospital stay. Data were expressed in the median with interquartile range, or number with percentage, where applicable. The patients admitted into ICU from PACU were compared to those admitted from the ward by using Mann–Whitney U test with Bonferroni correction or chi-square test where appropriate. *P* < 0.05 was considered statistically significant.

## Results

During the study period, 43,553 patients underwent general anesthesia and were admitted to the PACU. Among these patients, 109 patients (0.25%) underwent unplanned ICU admission directly from the PACU or subsequently from the ward: seventy-three from the PACU (0.17%) and thirty-six from the ward (0.08%). Both groups were similar in terms of patient characteristics including preoperative physical status and comorbidities (Table 1). Surgical and anesthesia procedures; and operative and anesthesia time were also similar between the groups, although the length of PACU stay was significantly shorter in the Ward group than in the PACU group (*p*<0.01) (Table 2).

**Table 1:** Patient characteristics

	Number (%) or Median (interquartile range)	
	PACU group (*n*=73)	Ward group (*n*= 36)
**Gender**		
Male	38 (51%)	21 (58%)
**Age (years)**	70 (58–77)	70 (60–78)
20–40	5 (6.8%)	1 (2.8%)
41–60	16 (21.9%)	8 (22.2%)
61–80	45 (61.7%)	22 (61.1%)
> 80	7 (9.6%)	5 (13.9%)
**Height (cm)**	159 (152–167)	162 (159–169)
**Weight (kg)**	56 (48–74)	57 (49–62)
**BMI (kg/m2)**	22.4 (20.1–26.1)	21.2 (19.8–24.1)
**ASA-PS**		
I	14 (19.2%)	3 (8.3%)
II	48 (65.8%)	24 (66.7%)
III	11 (15.0%)	9 (25.0%)
**Preoperative comorbidities**		
Hypertension	36	17
Coronary artery disease	8	5
Chronic heart failure	2	2
Arrhythmia	6	5
Peripheral artery disease	2	2
Diabetes Mellitus	17	9
Chronic kidney disease	10	9
Hemodialysis	4	6
Cerebral infarction	6	3
Seizure	2	1

Some of the comorbidities were overlapped

**Table 2:** Characteristics of surgical procedures and anesthesia care

	Number or Median (% or interquartile range)	
	PACU group (*n*=73)	Ward group (*n*=36)
**Emergency Surgery**	12 (16%)	6 (17%)
**Surgical Procedures**		
Neurosurgery	1	2
Head and neck surgery	11	1
Thoracic surgery	4	0
Gastrointestinal surgery	14	12
Gynecological surgery	7	1
Urology	12	6
Bone, joint, limb surgery	14	6
Spine surgery	5	3
Vascular surgery	1	1
Others	4	4
**Operation time (min)**	199 (112–280)	182 (119–328)
**General Anesthesia**		
Sevoflurane or Desflurane	69 (95%)	33 (92%)
with EDB or PNB	32 (44%)	16 (44%)
TIVA	4 (5%)	3 (8%)
with EDB or PNB	2 (3%)	1 (3%)
**Anesthesia time (min)**	281 (179–372)	272 (179–417)
**PACU time (min)**	89 (52–117)	52 (30–74)*

*PACU* Post-anesthesia care unit, *EDB* Epidural block, *PNB* Peripheral nerve block, *TIVA* Total intravenous anesthesia

^*^p < 0.01 vs. PACU group

**Fig. 1:**
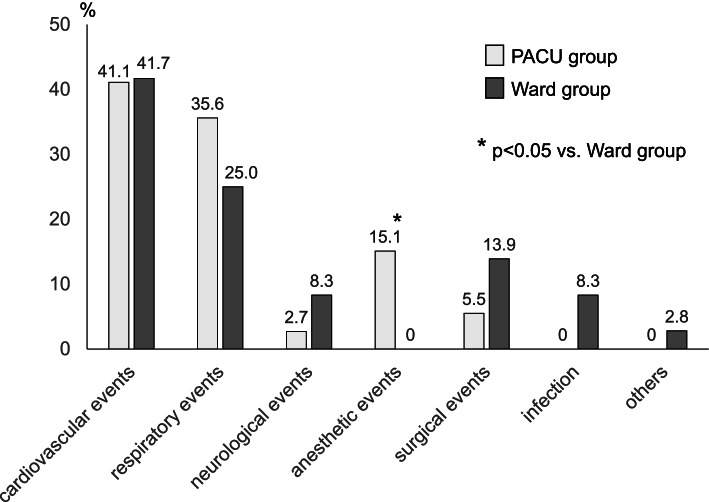
Percentage of adverse events leading to unplanned ICU admission in each group. The most frequent adverse events leading to unplanned ICU admission were cardiovascular events followed by respiratory ones. Anesthetic events were specific to the PACU group

The variation of the adverse events was comparable between the groups except for anesthetic events including delayed emergence and postanesthetic agitation which were more frequent in the PACU group. The most frequent adverse events leading to unplanned ICU admission were cardiovascular events followed by respiratory ones (Fig. 1), which was common among the groups. Hemodynamic instability including both hypotension and hypertension which required vasoactive and/or inotropic agents was the major cardiovascular event (Table 3). Concerning respiratory events, hypoxia was the most frequent adverse event (Table 3), although there was no difference in the number of patients who needed re-intubation or mechanical ventilation including non-invasive positive pressure ventilation (NPPV) between the groups (Table 4). The implementation of major interventions for the adverse events including mechanical ventilation, temporary hemodialysis, and circulation support such as extracorporeal membrane oxygenation (ECMO) was more frequent in the Ward group than in the PACU group: 13 interventions for the PACU group and 16 for the Ward group, respectively (*p*<0.01) (Table 4). Although twenty-two patients in the Ward group (61.1%) were admitted to the ICU within 3 days after discharge from the PACU (Table 5), there was no relationship between ICU admission and preoperative comorbidities or surgical procedures of each patient except for 3 cases of surgical bleeding. Cardiovascular events occurred most frequently during postoperative day (POD) 1 to 3, which occupied 28% (10 events) of the events in the Ward group (Table 5).

**Table 3:** Adverse events leading to unplanned ICU admission

		PACU group (*n*=73)	Ward group (*n*=36)
**Cardiovascular events**	hypotension	13	4
	hypertension	6	0
	arrhythmia	4	1
	heart failure	2	4
	myocardial ischemia	3	2
	pulmonary embolism	0	3
	cardiac arrest	1	1
	anaphylaxis	1	0
**Respiratory events**	hypoxia	21	5
	hypercapnia	1	0
	pneumothorax	1	1
	pneumonia	0	2
	airway obstruction	3	1
**Neurological events**	stroke	1	1
	involuntary movement	1	0
	seizure	0	2
**Anesthetic events**	delayed emergence	9	0
	agitation	2	0
**Surgical events**	surgical site bleeding	4	3
	anastomotic leakage	0	2
**Infection**	sepsis	0	3
**Others**	gastrointestinal hemorrhage	0	1

**Table 4:** Treatments for adverse events in ICU ^a^

		PACU group (*n*=73)	Ward group (*n*=36)
**Invasive intervention**	mechanical ventilation^b^	7	7
	NPPV	5	4
	HFNC	3	3
	hemodialysis	1	4
	ECMO	0	1
	reoperation	0	2
	others	2	2
**Medication**	vasoactive/inotropic drugs	12	7
	antiarrhythmics	4	0
	diuretics	1	4
	sedatives	2	0
	anticoagulants	0	3
	antibiotics	0	4
	others	2	2
**Transfusion**	RBC	3	1
	FFP	3	0

*NPPV* Non-invasive positive pressure ventilation, *HFNC* High-flow nasal cannula, *ECMO* Extracorporeal membrane oxygenation, *RBC* Packed red blood cells, *FFP* fresh frozen plasma

^a^ Some of the treatments are overlapped

^b^ Accompanied by re-intubation

**Table 5:** Time point of ICU admission and adverse events in Ward group

		< POD 1	POD 1–3	> POD 3
**Cardiovascular events**	hypotension without definite diagnosis	1	1	2
	arrhythmia		1	
	congestive heart failure		3 (2)	1 (1)
	myocardial ischemia		1 (1)	1
	pulmonary embolism		3 (1)	
	cardiac arrest		1 (1)	
**Respiratory events**	atelectasis	2	2	1 (1)
	pneumothorax			1
	pneumonia			2 (1)
	laryngeal edema			1
**Neurological events**	stroke		1	
	seizure		1	1
**Surgical events**	surgical site bleeding	1 (1)	2 (2)	
	anastomotic leakage			2 (2)
**Infection**	sepsis		1 (1)	2 (1)
**Others**	gastrointestinal bleeding		1 (1)	

(): number of patients who received major interventions including mechanical ventilation, circulation support, hemodialysis, or re-operation. *POD* Postoperative day

The length of ICU stay was significantly longer in the Ward group than in the PACU group (*p*<0.01). Almost 95% of the patients in the PACU group were discharged from the ICU within 2 days after the admission, although 47% of those in the Ward group stayed in the ICU for more than two days (Table 6). The percentage of patients who stayed in the hospital for longer than 30 days after surgery was significantly higher in the Ward group than in the PACU group (27.4% vs. 58.3%, *p*<0.01); accordingly, the length of hospital stay was also longer in the Ward group than in the PACU group (*p*<0.01) (Table 6). Nevertheless, there was no significant difference in in-hospital mortality between the groups (4.1% vs. 8.3%, respectively) (Table 6).

**Table 6:** Length of ICU and hospital stay, in-hospital mortality

		Median (interquartile) or Number (%)	
		PACU group	Ward group
Length of ICU stay (days)	Median	1.4 (1.4–1.5)	2.5 (1.5–3.5)*
	1–2 days	69 (94.5%)	19 (52.8%)*
	≥ 3 days	4 (5.5%)	17 (47.2%)*
Hospital stay >30 days after surgery		20 (27.4%)	21 (58.3%)*
Length of hospital stay (days)		19 (10–40)	39 (18–89)*
In-hospital mortality		3 (4.1%)	3 (8.3%)*

^*^p < 0.01 vs. PACU group

## Discussion

In this retrospective study, we examined the clinical characteristics of patients who experienced unplanned ICU admission from the PACU and the ward. Several studies have investigated the clinical characteristics or outcomes of unplanned ICU admission after surgery [1, 2]; however, our study focused on the comparison of clinical features between direct admission from the PACU and delayed admission from the general ward.

The overall incidence of unplanned ICU admission after general anesthesia in our institute was 0.25%, which was relatively low considering the results of previous reports ranging from 0.28% to 2.2% [4–7]. The variation in the incidence of unplanned ICU admission among the previous studies may be attributed to the difference in the definition of unplanned ICU admission, inclusion criteria, or institutional practice. In our study, 67% of the patients who experienced unplanned ICU admission had adverse events in the PACU and 33% in the ward, with no statistical difference. Cardiovascular and respiratory events were the most frequent adverse events in both groups (Fig. 1); hypoxia and hypotension accounted for 40% of all adverse events. These events seemed more frequent in the PACU group although there was no statistical difference (Table 3). This tendency might be attributed to the anesthesia-related factors such as residual effects of anesthetic agents considering delayed emergence and agitation were specific to the PACU group and accounted for 10% of all adverse events. Haller and colleagues described hypotension and persistent oxygen desaturation accounted for about 40% of incidences leading to unplanned ICU admission, which was similar to the results of our study [1]. Rujirojindakul and colleagues found 164 cases of re-intubation in the PACU out of 117,502 patients undergoing general anesthesia (0.14%) [8], although the overall incidence of re-intubation in our study was 0.03% with no difference between the groups. This low incidence of re-intubation might be partially attributed to the implementation of NPPV or high-flow nasal cannula, although the incidence of respiratory support including these therapies was as low as 0.06%; hence, we consider the incidence of respiratory support in this study was clinically acceptable.

However, the major interventions including mechanical ventilation (invasive or non-invasive), temporary hemodialysis, and ECMO were more frequently implemented in the Ward group than in the PACU group. And the length of both ICU and hospital stay was significantly longer in the Ward group. Notably, 95% of the patients in the PACU group were discharged from the ICU within 2 days after admission, although almost half of those in the Ward group stayed in the ICU for more than 2 days. Considering these results and a trend of higher in-hospital mortality in the Ward group, it could be considered the patients in the Ward group suffered from more severe complications than in the PACU group. Interestingly, the length of PACU stay was significantly shorter in the Ward group, which may indicate the Ward group was as stable as they could satisfy the discharge criteria of the PACU. In contrast, the longer length of PACU stay in the other group might reflect the exertion to manage the adverse events which were beyond the level of care in the PACU. A previous study, in which the influence of the length of PACU stay on clinical outcomes was examined, indicated prolonged PACU stay was associated with a higher incidence of clinical deterioration in the ward within 24 h after discharge from PACU [9]. These results may suggest prompt decision-making for ICU admission would be crucial if a certain level of intervention or intensive monitoring is necessary in the PACU. Actually, 95% of patients in the PACU group were discharged from the ICU within 2 days. Concerning the adverse events in the Ward group, the cardiovascular ones, which were not directly related to the preoperative comorbidities or surgical procedures, occupied 41.7% of the events. And the patients with cardiovascular events, especially during POD 1 to 3, were prone to receive major interventions in the ICU. As it would have been difficult to predict these events from preoperative comorbidities or surgical procedures in each patient, postoperative patient evaluation according to certain evaluation criteria such as Clavien-Dindo classification or modified one in the ward within 3 postoperative cardiovascular complications. Early detection of postoperative complications would be crucial because unplanned ICU admission from the ward has been also shown associated with increased emergency hospital readmission after surgery [10].

Unplanned ICU admission is associated with increased morbidity and mortality [11, 12], and has been shown to be an important safety measure of anesthesia and surgical care [1, 2]. Gillies and colleagues demonstrated higher mortality and greater requirement for organ supports in patients with delayed admission to the ICU after initial ward care, compared to those directly admitted to the ICU from the recovery room or in their retrospective study which included more than 500,000 surgical patients [12]. Although we could not show a significant difference in mortality between the groups, we consider a trend of higher in-hospital mortality in the Ward group does not contradict the results of the work by Gillies.

It is eligible to identify risk factors for unplanned ICU admission to improve patient safety and outcome. Previous studies have indicated several predictive factors for postoperative unplanned ICU admission such as higher ASA-PS, advanced age, elevated BMI, and prolonged surgical procedure [1, 13, 14]. However, we could not identify either a difference in patient characteristics between the groups or a specific factor related to unplanned ICU admission in this study. Because high-risk patients evaluated as ASA-PS 4 or higher were scheduled for the postoperative ICU admission in our institute, the patients undergoing unplanned ICU admission were evaluated as ASA-PS 3 or less in this study, although the previous ones included the higher ASA-PS. Moreover, our patients were generally thinner than those in the previous studies, and only a few patients presented BMI of more than thirty in this study group. These patient characteristics might partly explain why we could not find a predictive factor associated with unplanned ICU admission in this study, however, there could be a certain probability of adverse events independent of patient characteristics or surgical procedures.

As this was a retrospective study, the contribution of the PACU to the improvement of the patient outcome could not be determined directly. However, early detection of the adverse events and prompt intervention would at least work well to ameliorate the symptoms. A prospective large-scale study would be eligible to prove the significance of the PACU to improve patient outcomes. Furthermore, a bias of the patient selection for ICU admission might have affected the clinical course after the ICU admission. The ICU admission is usually determined by the anesthesia team in the PACU and by the surgical team in the ward. And the threshold of decision for the ICU admission in the PACU group could have been lower than in the Ward group because the anesthesia team is more familiar with patient care in the ICU than the surgical team, which may partly explain the better outcome in the PACU group. However, this bias could be common in the previous studies because the PACU is managed by the anesthesiologists in most institutions and decision-making for ICU admission is dependent on case-by-case consideration rather than definite criteria. Thus, it is speculated this bias did not affect the interpretation of the results in this study. In contrast to the previous studies that indicated preoperative or operative risk factors for unplanned ICU admission [1, 13, 14], we could not find specific risk factors in this study. Although the size of the study population was not too small compared to the previous ones, a relatively low incidence of unplanned ICU admission might have affected the analysis of predictive factors for unplanned ICU admission from the ward. However, at least the results of this study indicated that delayed ICU admission was related to the need for major interventions and prolonged both ICU and hospital stay.

## Conclusions

The incidence of unplanned ICU admission after PACU stay was low in this single-center, retrospective, observational study. However, delayed admission to the ICU from the ward prolonged the length of both ICU and hospital stay compared to direct admission from the PACU. Early recognition of the adverse events and prompt decision-making for ICU admission might be associated with the shortening of ICU and/or hospital length of stay. A future prospective study would elucidate the importance of appropriate monitoring and prompt decision-making for ICU admission in the PACU would improve patient outcomes.

## Data Availability

The datasets generated and/or analyzed during the current study are not publicly available because the institutional rules strictly prohibit releasing the native data on the web but are available from the corresponding author on reasonable request.

## Abbreviations

ICU: Intensive care unit
PACU: Post-anesthesia care unit
OR: Operating room
ASA-PS: American Society of Anesthesiologists physical status
BMI: Body mass index
NPPV: Non-invasive positive pressure ventilation
ECMO: Extracorporeal membrane oxygenation

## References

[1] Haller G, Myles PS, Wolfe R, Weeks AM, Stoelwinder J, McNeil J (2005). Validity of unplanned admission to an intensive care unit as a measure of patient safety in surgical patients. Anesthesiology.

[2] Haller G, Myles PS, Langley M, Stoelwinder J, McNeil J (2008). Assessment of unplanned admission to the intensive care unit as a global safety indicator in surgical patients. Anaesth Intensive Care.

[3] Aldrete JA (1995). The post-anesthesia recovery score revisited. J Clin Anesth.

[4] Jhanji S, Thomas B, Ely A, Watson D, Hinds CJ, Pearse RM (2008). Mortality and utilisation of critical care resources amongst high-risk surgical patients in a large NHS trust. Anaesthesia.

[5] Pearse RM, Moreno RP, Bauer P, Pelosi P, Metnitz P, Spies C, Valle Bt, Vincent JL, Hoeft A, Rhodes A (2012). Mortality after surgery in Europe: a 7 day cohort study. Lancet.

[6] Meziane M, Jaouhari SDE, ElKoundi A, Bensghir M, Baba H, Ahtil R, Aboulaala K, Balkhi H, Haimeur C (2017). Unplanned intensive care unit admission following elective surgical adverse events: incidence, patient characteristics, preventability, and outcome. Indian J Crit Care Med.

[7] Cullen DJ, Nemeskal AR, Cooper JB, Zaslavsky A, Dwyer MJ (1992). Effect of pulse oximetry, age, and ASA physical status on the frequency of patients admitted unexpectedly to a postoperative Intensive Care Unit and the severity of their anesthesia-related complications. Anesth Analg.

[8] Rujirojindakul P, Geater AF, McNeil EB, Vasinanukorn P, Prathep S, Asim W, Naklongdee J (2012). Risk factors for reintubation in the post-anaesthetic care unit: a case-control study. Br J Anaesth.

[9] Mann-Farrar J, Egan E, Higgins A, Wysocki L, Vaux A, Arndell E, Burmeister EA (2019). Are postoperative clinical outcomes influenced by length of stay in the postanesthesia care unit?. J Perianesth Nurs.

[10] Gillies MA, Ghaffar S, Harrison E, Haddow C, Smyth L, Walsh TS, Pearse RM, Lone NI (2019). The association between ICU admission and emergency hospital readmission following emergency general surgery. J Intensive Care Soc.

[11] Piercy M, Lau S, Loh E, Reid D, Santamaria J, Mackay P (2006). Unplanned admission to the intensive care unit in postoperative patients–an indicator of quality of anaesthetic care?. Anaesth Intensive Care.

[12] Gillies MA, Harrison EM, Pearse RM, Garrioch S, Haddow C, Smyth L, Parks R, Walsh TS, Lone NI (2017). Intensive care utilization and outcomes after high-risk surgery in Scotland: a population-based cohort study. Br J Anaesth.

[13] Quinn TD, Gabriel RA, Dutton RP, Urman RD (2017). Analysis of unplanned postoperative admissions to the Intensive Care Unit. J Intensive Care Med.

[14] Bruceta M, De Souza L, Carr ZJ, Bonavia A, Kunselman AR, Karamchandani K (2020). Post-operative intensive care unit admission after elective non-cardiac surgery: a single-center analysis of the NSQIP database. Acta Anaesthesiol Scand.

